# Fast modification on wheat straw outer surface by water vapor plasma and its application on composite material

**DOI:** 10.1038/s41598-018-20285-5

**Published:** 2018-02-02

**Authors:** Weimin Chen, Yicheng Xu, Shukai Shi, Yizhong Cao, Minzhi Chen, Xiaoyan Zhou

**Affiliations:** 1 College of Materials Science and Engineering, Nanjing Forestry University, Nanjing, 210037 China; 2 Department of Civil and Environment Engineering, University of Alberta, Edmonton, T6G 2W2 Canada; 3 Jiangsu Engineering Research Center of Fast-growing Trees and Agri-fiber Materials, Nanjing, 210037 China; 4 Nanjing Suman Plasma Technology Co., Ltd, Enterprise of Graduate Research Station of Jiangsu Province, No. 3 Youyihe Road, Nanjing, 210001 China

## Abstract

The presence of non-poplar extracts, cutin, and wax layer in the wheat straw outer surface (WOS) greatly limit its application in bio-composite preparation. In this study, a dielectric-barrier-discharge plasma using water vapor as feeding gas was used to fast modify the WOS. The morphology, free radical concentrations, surface chemical components, and contact angles of WOS before and after plasma modification were investigated. Wheat straw was further prepared into wheat straw-based composites (WSC) and its bonding strength was evaluated by a paper tension meter. The results showed that water vapor plasma leads to the appearance of surface roughness, the generation of massive free radicals, and the introduction of oxygen-containing groups. In addition, both initial and equilibrium contact angle and the surface total free energy were significantly increased after plasma modification. These results synergistically facilitate the spread and permeation of adhesive onto the WOS and thus improve the bonding strength of all prepared WSCs. A good linear relationship between bonding strength and surface roughness parameters, contact angles, and total free energy were observed. In general, this study provided a time-saving and cost-effective modification method to realize WSC manufacture.

## Introduction

Annual outputs of wheat, corn, and rice straw reached approximately 600 million tons in China. Especially, wheat straw accounts for the majority in the northwest area. Most of the wheat straws were directly burned, which resulted in air pollution issues. Only a small part of wheat straws was further applied in bio-energy conversion and bio-composite manufacture^1^. However, the presence of non-poplar extracts, cutin, and wax layer in the wheat straw outer surface (WOS) greatly limits its application in bio-composite manufacture since the adhesive is difficult to spread and permeate^2–4^. In addition, wheat straw has lower contents of cellulose and lignin, nevertheless higher pentosan, which also leads to poor bonding strength^1,4^. Therefore, modification on WOS is necessary to realize its industrialization on bio-composite manufacture.

Steam explosion^1,5^, hydrothermal pretreatment^6^, ozonolysis^7^, and extraction using ethanol/benzene^8^ have been applied in previous studies to effectively remove the cutin and wax layer in WOS for higher yields of sugar and ethanol. Meanwhile, these methods also lead to the significant improvement on bonding strength for the preparation of paper products or panels. However, these methods are energy-consuming and time-consuming. In particular, steam explosion requires pre-soaking process (at 20 °C, for 12 hours) and heat treatment (at 200 °C, for 2–3 min)^1,5^; Extraction method requires the extracting process using ethanol/benzene solution for 24 hours^8^. In addition, the bulk properties of wheat straw may be also affected. A more effective modification method on WOS should be focused on enhancing the bonding strength of bio-composites and simultaneously declining the production cost.

In recent years, plasma technology has been considered as an attractive modification method on material’s surface. Compared to other conventional methods, modification by plasma is a solvent-free and non-polluted method which requires short processing period^9^. The conventional methods change the bulk properties of the material; however, plasma only impacts the surface properties^10^. In addition, plasma makes it possible to introduce various target chemical groups into material’s surface via simply changing the feeding gas type (O_2_, NH_3_, air, N_2_, etc)^11–13^. To achieve the target properties of material surface such as hydrophilicity, previous studies have applied the commonly used radio frequency (RF) plasma^14–16^. RF plasma requires a high vacuum condition which increases the modification cost associated with the power consumption and capital cost^17,18^. However, dielectric barrier discharge (DBD) plasma has become a promising modification method since it can work under atmospheric pressure^19–22^. In addition, more reactive oxidizing species and uniform discharge can be generated as compared to the RF plasma. DBD plasma leads to less mass loss of material’s surface during the modifying process^23^. Our previous work has demonstrated that DBD plasma using glycidyl methacrylate as feeding gas can significantly improve the bonding strength of wheat straw-based composite (WSC) from 0.034 MPa to 1.049 MPa^24^. However, due to toxicity and the high cost of glycidyl methacrylate, it is recommended to use a non-toxic, and cheap feeding gas such as water vapor^25^. As one of the effective parameters on bonding strength of WSC, plasma discharge power can affect the generations of free radicals and target chemical groups. To the best of our knowledge, there is little information about the relationship between plasma discharge power and bonding strength of WSC.

The aim of this study is to find a time-saving and cost-effective modification method to improve the bonding strength of WSC. To achieve this purpose, a self-designed DBD plasma modifying system will be developed. Water vapor as a non-toxic and low-cost compound will be used as feeding gas to investigate its ability to generate various polar oxygen-containing groups under different plasma discharge power. In addition, the effects of plasma discharge power on physicochemical properties of WOS will be studied via a series of characterization methods to find the relation between plasma discharge power and bonding strength of WSC.

## Materials and Methods

### Raw materials

The wheat straw used in this study was purchased from a local factory. It was washed using distilled water to remove any impurity and cut into pieces (50 × 5 mm) followed by drying in an oven at 103 °C for 6 hours.

Urea-formaldehyde resin (UF) was self-prepared in our lab. Phenol-formaldehyde resin (PF) was bought from Taier Chemical Industry Co. Ltd. The common physicochemical properties of these two adhesives are presented in Table 1.

**Table 1:** Basic properties of UF and PF adhesive.

	Solid content (%)	pH	Viscosity (mPa·s)	Density (g·cm^−3^)	Surface tension (mJ·m^−2^)
UF	55.1	7.4	128	1.12	84.9
PF	52.0	9.8	270	1.21	93.4

### Water vapor plasma modification

**Figure 1:**
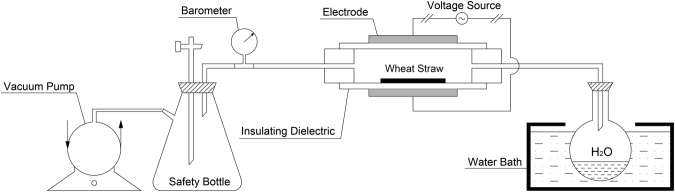
The schematic diagram of plasma modifying system.

The DBD plasma modifying system was self-designed in our lab as presented in Fig. 1. Prior to the experiment, the wheat straw was placed in the reacting chamber with outer surface upward. A vacuum pump was used to purge the system for 10 min with water vapor to remove the air. The plasma generator (CTP-2000K) was used to excite the water vapor inside the reacting chamber. Details of the modification setup were described elsewhere^24^. The experiment conditions used in the current study are listed as follows: modifying time, 120 s; system pressure, 20 KPa. Discharge power varies from 0, 30, 40, and 50 W. The corresponding untreated and modified wheat straws are denoted as UW, PW-30, PW-40, and PW-50, respectively.

### Characterization methods

The surface morphology of plasma modified wheat straw as well as untreated samples were studied by scanning electron microscope (SEM, JSM-7600F). Prior to imaging, the samples were subjected to gold coating to reduce the charging effect. Atomic force microscope (AFM) was used to further investigate the surface morphology. The detailed steps can be found in our previous study^24^. Briefly, wheat straw was processed into white fiber using a solution of hydrogen peroxide and glacial acetic acid. The mica sheet loaded with white fibers was attached to the observation stage for the imaging. The roughness parameters of *R*_*a*_ and *R*_*b*_ were determined based on the 2-dimensional (2D) section lines of WOS by applying the XEI software. Electron spin resonance spectrometry (ESR, Bruker EMX-10/12) was used to determine the concentration of free radicals. The testing parameters are listed as follows: microwave frequency, 9.8617 GHz; scan time period, 5 min; time constant, 0.25 s. X-ray photoelectron spectroscopy (XPS, AXIS Ultra DLD) was performed to investigate the surface chemical properties of the samples including the compositions of surface atoms and chemical groups. The survey-scan mode was applied to record the low-resolution spectra with a binding energy region of 0–1200 eV. In addition, the spectra of high-resolution (C1s) were recorded at the binding energy region of 277–296 eV. The C1s spectra were further deconvoluted into 4 Gaussian peaks which represent 4 carbon-related chemical groups (-C-C-/-C-H at 284.6 eV; -C-OH at 286.5 eV; -C=O at 287.8 eV; O=C-O- at 288.8 eV). The surface wettability of wheat straw was evaluated based on the contact angles including initial and equilibrium angles which were calculated by the optical contact angle measuring apparatus (Theta) and using UF or PF as testing drops. Distilled water was also used as testing drop to determine the surface free energy of polar components via the method of Owens-Wendt^24^, while diiodomethane was used for the calculation of the surface free energy of non-polar components. The attenuation coefficient (K) was defined as the varied rate of contact angles with spreading time increased. The related calculating equation can be referred to our previous study^24^.

### Preparation of WSC and its bonding strength

WSC was assembled via the sandwich-like form. UF and PF were used as adhesives coated in the interface of two wheat straw surface. Then, the assembled composite was dried in an oven at 80 °C for 4 h. Subsequently, it was subjected to a hot pressing process in a manual press machine to obtain the final WSC. The detailed hot pressing conditions of the WSC using UF and PF adhesives are listed in Table 2. A paper tension meter (WZL-300) was applied to determine the bonding strength of WSC based on the testing standard of ISO1924/2-1985. The detailed testing steps can be referred to our previous study^26^.

**Table 2:** Hot pressing conditions with UF and PF adhesives.

Hot pressing conditions	UF	PF
Adhesive content for single outer side	200 g·cm^−3^	200 g·cm^−3^
Bonding section	15 × 5 mm	15 × 5 mm
Hot pressing temperature	110 °C	130 °C
Squeezing pressure	1.5 MPa	1.5 MPa
Squeezing time	90 s	140 s

## Results and Discussion

### Surface morphology

**Figure 2:**
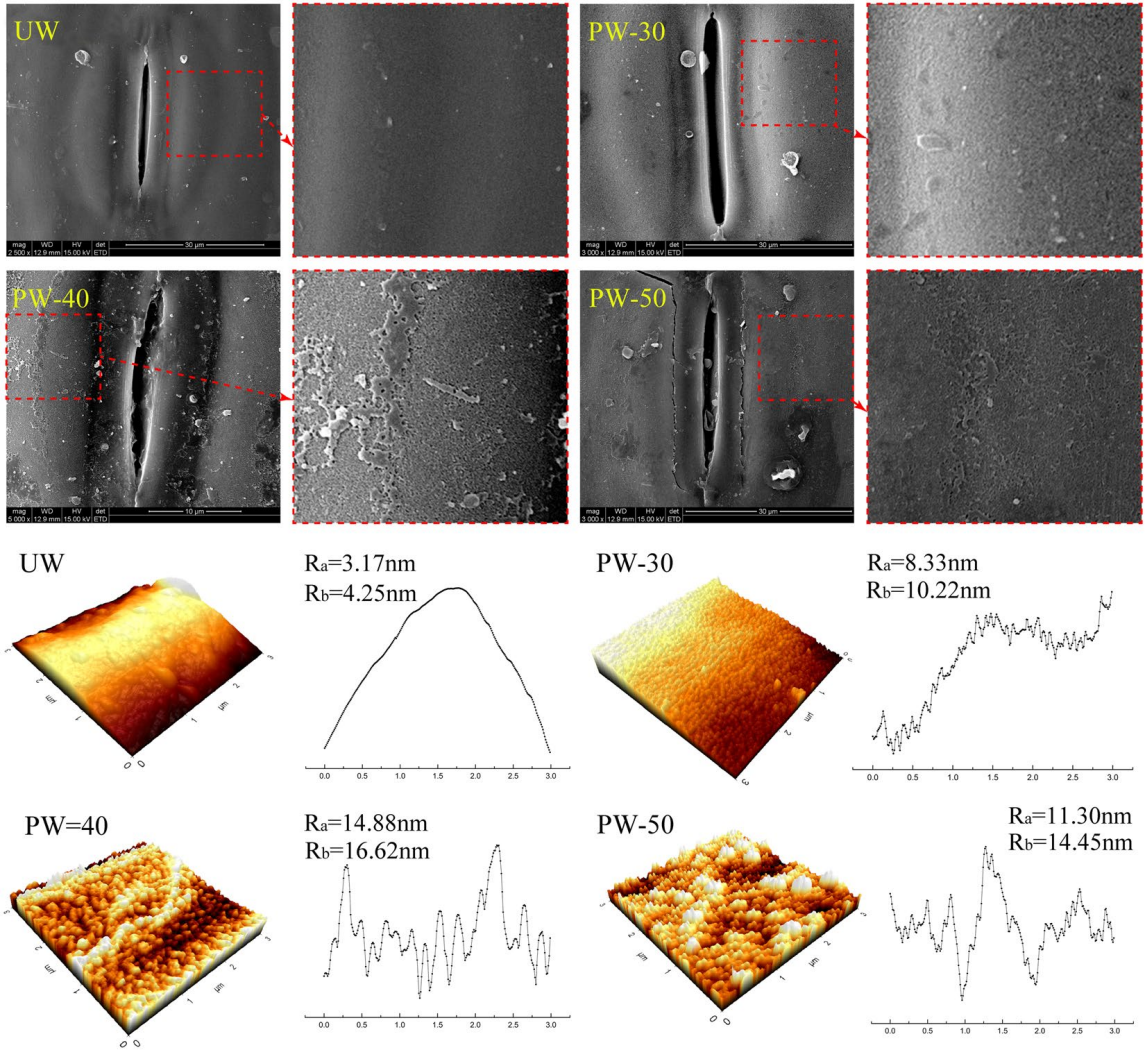
Surface morphology of untreated wheat straw and plasma treated samples.

Figure 2 shows the SEM images of the outer surface for all the samples. A smooth outer surface was observed on the UW. However, after plasma modification, some bulges appear on the PW-30. The PW-40 demonstrates surface roughness with continuous bulges leading to the formation of stratified structure. These phenomena can be attributed to the etching effect of plasma which is caused by the bombardment of water vapor-excited ions/atoms with high energy^27^. Further increase of plasma discharge power to 50 W results in the formation of two obvious cracks on the stratified structure. This can be explained by the excessive plasma discharge power leading to the self-degradation and gasification of outer surface, consequently resulting in the surface shrinkage and the formation of cracks^19^. Further investigation on the morphology of outer surface of the samples was carried out using AFM as shown in Fig. 2. A smooth surface is also observed on the UW, which is in good agreement with the SEM analysis. However, the PW-30 demonstrates a hill-like structure on the outer surface. Increasing the plasma discharge power to 40 W, a popcorn-like structure is observed. This structure became intensified when plasma discharge power was increased further to 50 W. The modified samples suggest a favorable surface structure for the adhesives to spread and permeate onto the outer surface and to form a nail-like adhesive in the interface of WSC.

The 2D section lines and the parameters of surface roughness are demonstrated in Fig. 2. Both *R*_*a*_ and *R*_*b*_ are dramatically increased by increasing the plasma discharge power and show 369% and 291% growth on the PW-40 compared to the UW, respectively. However, a decrease for both roughness parameters (*R*_*a*_ and *R*_*b*_) is observed on the PW-50 compared to the PW-40. This result can be attributed to the gasification of outer surface at excessive discharge power.

### Surface chemical properties

The free radicals concentration of wheat straw samples was determined by ESR as presented in Table 3. An increasing trend is observed with the plasma discharge power increased. Especially, the free radicals concentration of the PW-50 reaches 2.5 × 10^16^ spin·g^−1^ which is 210% higher than that of the UW. This observation can be related to the breakage of raw chemical bonds in wheat straw surface and the excitation of water vapor. The increase in the free radicals concentration after plasma modification indicates a possibility of chemical reactions in outer surface.

**Table 3:** The surface properties of untreated wheat straw and plasma treated samples (T., Surface total free energy; P., Surface free energy of polar components; D., Surface free energy of non-polar components).

	Carbon-related chemical groups (%)				Atomic components (%)			Surface free energy (mJ/m^2^)			Radical concentration (×10^16^ spin/g)
	-C-C-	-C-OH	-C=O	-O-C=O	C	O	Si	T.	P.	N.	
UW	84.5	10.2	3.7	1.6	91.0	7.4	0.7	34.7	7.3	27.4	0.8 ± 0.1
PW-30	71.8	21.5	4.9	1.8	82.3	11.4	5.8	45.8	16.6	29.2	1.8 ± 0.2
PW-40	67.0	18.1	6.0	8.9	68.5	26.5	4.4	57.2	33.3	23.9	2.4 ± 0.2
PW-50	52.7	24.0	8.0	15.3	65.0	28.6	5.5	50.9	26.9	24.0	2.5 ± 0.2

**Figure 3:**
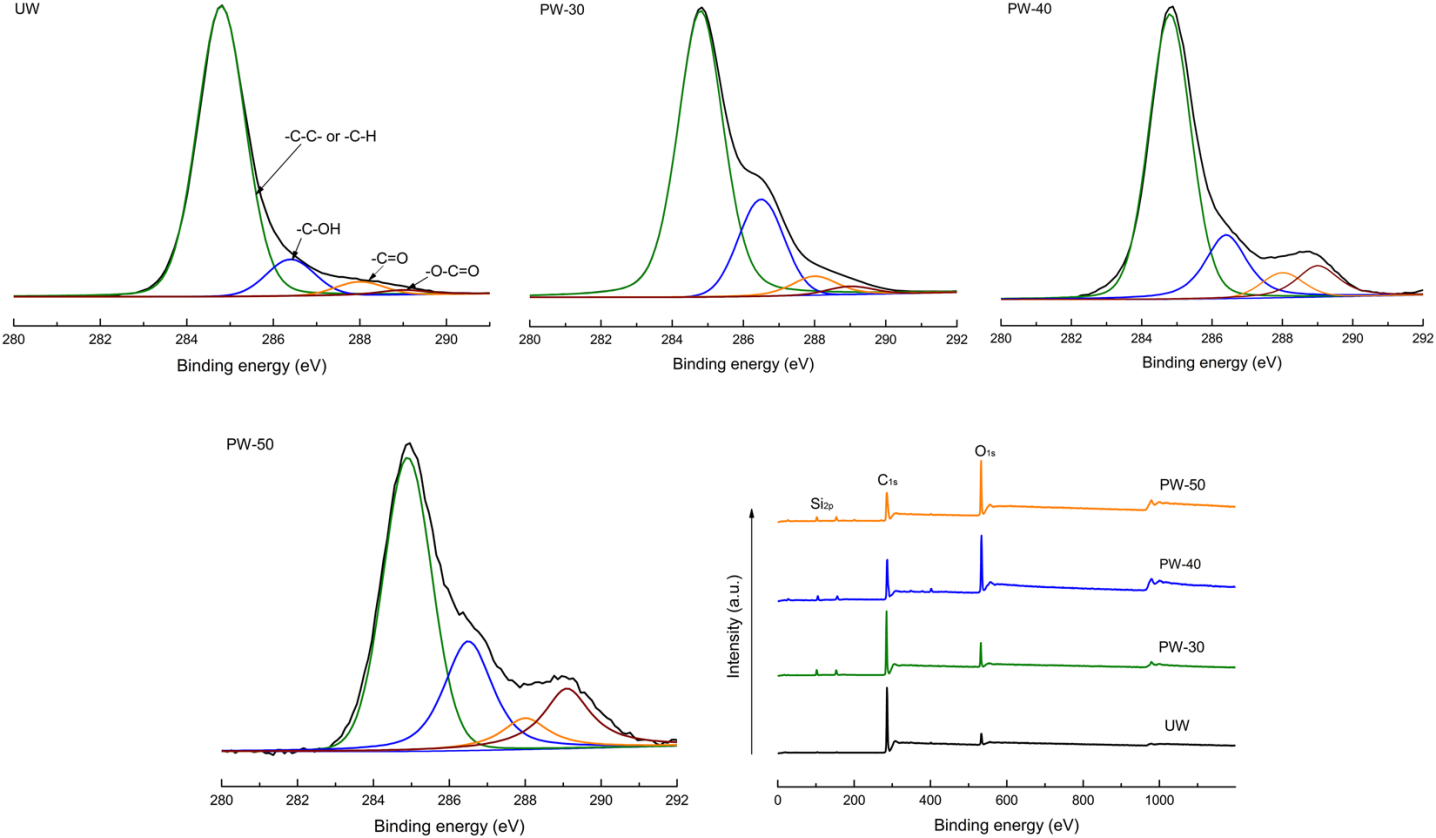
XPS spectra of untreated wheat straw and plasma treated samples.

To confirm the existence of surface chemical reactions, XPS analysis was used in this study and the results are presented in Fig. 3 and Table 3. It can be observed from Fig. 3 that the Si signals appear after plasma modification showing the presence of Si element on the outer surface. The etching effect of plasma leads to the generation of bulges and stratified structure (observed by SEM images) which therefore lead to the exposure of Si element^28^. The oxygen content progressively increased with the plasma discharge power increased. In addition, oxygen-containing chemical groups also show an increasing trend particularly for the O-C=O bond which demonstrates significantly increase from 1.6% (UW) to 15.3% (PW-50). Plasma modification can excite the water vapor and consequently generate several oxygen-containing fragments with active sites^20^. The excited ions and atoms with high energy from plasma modification are bombarded onto the outer surface, which results in the breakage of raw chemical bonds and the generation of various active sites. The reactions between the oxygen-containing fragments and free active sites in outer surface lead to the introduction of stable oxygen-containing chemical groups. In addition, the significant decrease in the content of -C-C- from 91.0% (UW) to 65.0% (PW-50) also gives an evidence that ions and atoms bombardment results in the breakage of skeleton chemical bond in outer surface.

### Surface contact angle and free energy

**Figure 4:**
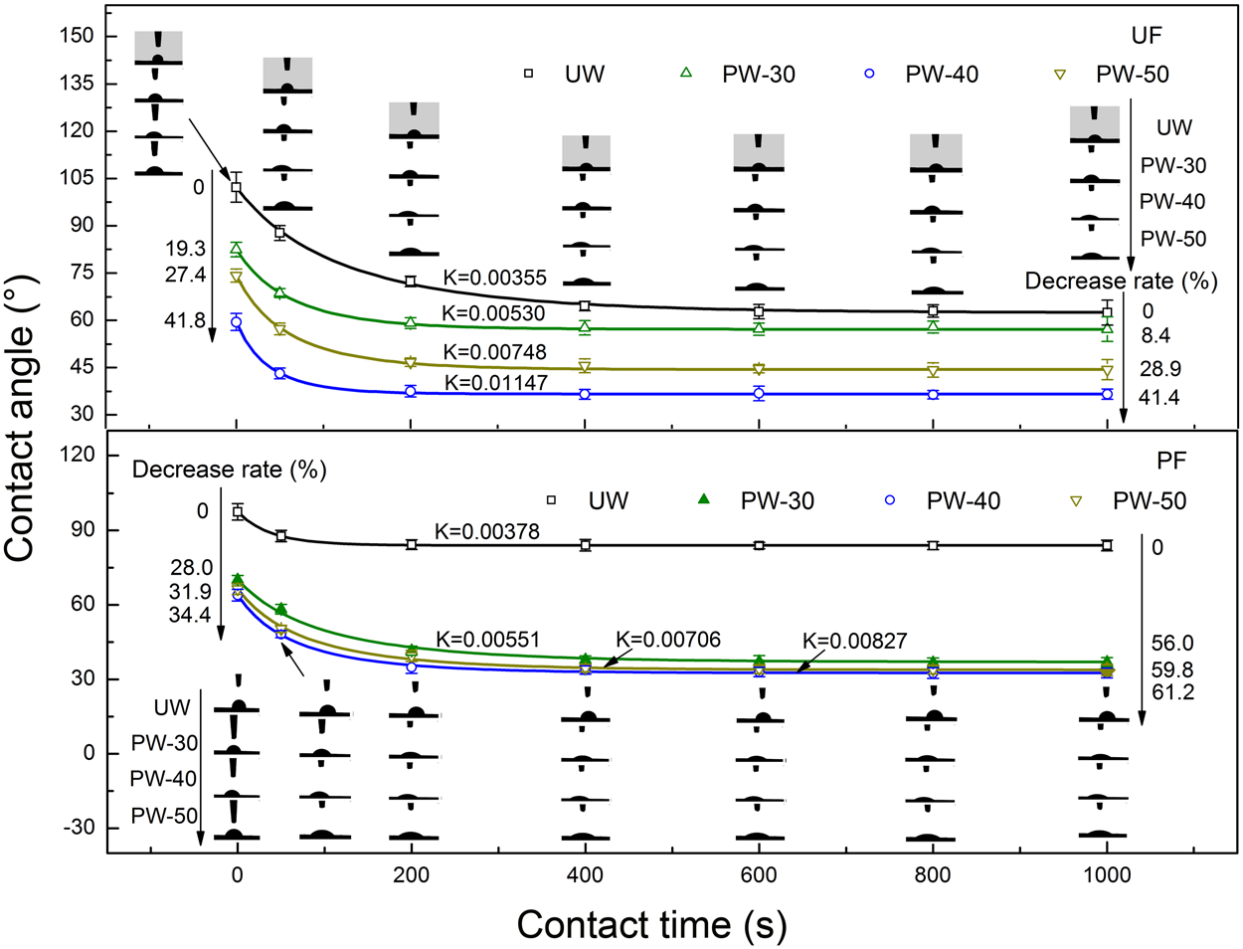
Contact angles varied with increasing contact time using the droplet of UF or PF.

Surface wettability greatly depends on the surface roughness and contact angle^19^. Since SEM and AFM analysis already proved that plasma modification increases the surface roughness of outer surface, surface contact angle should be further investigated to evaluate the surface wettability of the modified wheat straw. Figure 4 shows the surface contact angles including initial and equilibrium angles versus contact time. For the testing drop of UF adhesive, the maximum decreases of 41.8% (initial angle) and 41.4% (equilibrium angle) are observed on the PW-40 in comparison to the UW. Plasma modification develops roughness on the outer surface and forms a popcorn-like structure which greatly promotes the permeation of adhesive. In addition, the introduction of oxygen-containing groups such as carboxyl on the outer surface facilitates the spreading of adhesive. These two effects synergistically decrease the surface contact angle of outer surface. For the testing drop of PF adhesive, the same variation trend is observed.

Surface free energy as an important evaluating factor on surface wettability was calculated based on the value of contact angles. The results are listed in Table 3. It can be concluded that plasma modification increases surface total free energy. The maximum value of 57.2 mJ/m^2^ is observed on the PW-40, which is 64.9% higher than that of the UW, while the surface free energy of non-polar components is slightly decreased on the PW-40. This result implies that the increase in surface total free energy is mainly due to the increase in surface free energy of polar components, which also proves the introduction of oxygen-containing groups. It also can be observed from Table 3 that the PW-40 shows the highest value of attenuation coefficient (K) indicating that the surface of the PW-40 has the highest spreading and permeating rate for both UF and PF adhesive.

### Bonding strength of WSC

WSC was prepared via bonding two species of wheat straw surface using UF or PF as adhesive. The bonding strengths of the two types of WSCs (outer-outer and inter-inner) were investigated as presented in Table 4. ‘Outer-Outer’ in the Table 4 refers to the bio-composite whose adhesion interface was formed between two outer surfaces. Similarly, ‘Outer-Inner’ stands for the bio-composite whose adhesion interface was formed between an outer surface and an inner surface. WSC prepared by untreated wheat straw shows a very low bonding strength which cannot meet the basic requirements of the construction material. After plasma modification, the outer-outer bonding strengths of all the bio-composites are increased. Using the PF as adhesive, the bonding strength of the PW-40 achieves the maximum value of 0.441 MPa which is 549% higher than that of the untreated WSC. The enhancement in the bonding strength is attributed to the improved surface wettability and the introduction of oxygen-containing groups which can facilitate the spreading and permeating of adhesive onto the outer surface. In the case of outer-inner bonding strength, the highest value of 0.756 MPa is observed on the PW-40 which is higher than the outer-outer bonding strength, since the inner surface contains no cutin and wax layer blocking the contact between adhesive and wheat straw outer surface. This result also indicates that water vapor plasma modification is an effective method for the manufacture of WSC since the wheat straw can be randomly assembled whether in the form of outer-outer or outer-inner. It should be noted that WSC using PF as adhesive has generally higher bonding strength in comparison to that using UF as adhesive. This result is attributed to the inherent properties of PF such as high viscosity and cross-linked network structure after solidification.

**Table 4:** The bonding strength of WSCs.

	Outer-Outer (MPa)		Outer-Inner (MPa)	
	UF	PF	UF	PF
UW	0.034 ± 0.003	0.068 ± 0.013	0.092 ± 0.018	0.123 ± 0.024
PW-30	0.113 ± 0.031	0.340 ± 0.069	0.256 ± 0.059	0.523 ± 0.067
PW-40	0.230 ± 0.055	0.441 ± 0.056	0.467 ± 0.070	0.756 ± 0.109
PW-50	0.195 ± 0.041	0.375 ± 0.054	0.388 ± 0.048	0.613 ± 0.077

### Modification mechanism

**Figure 5:**
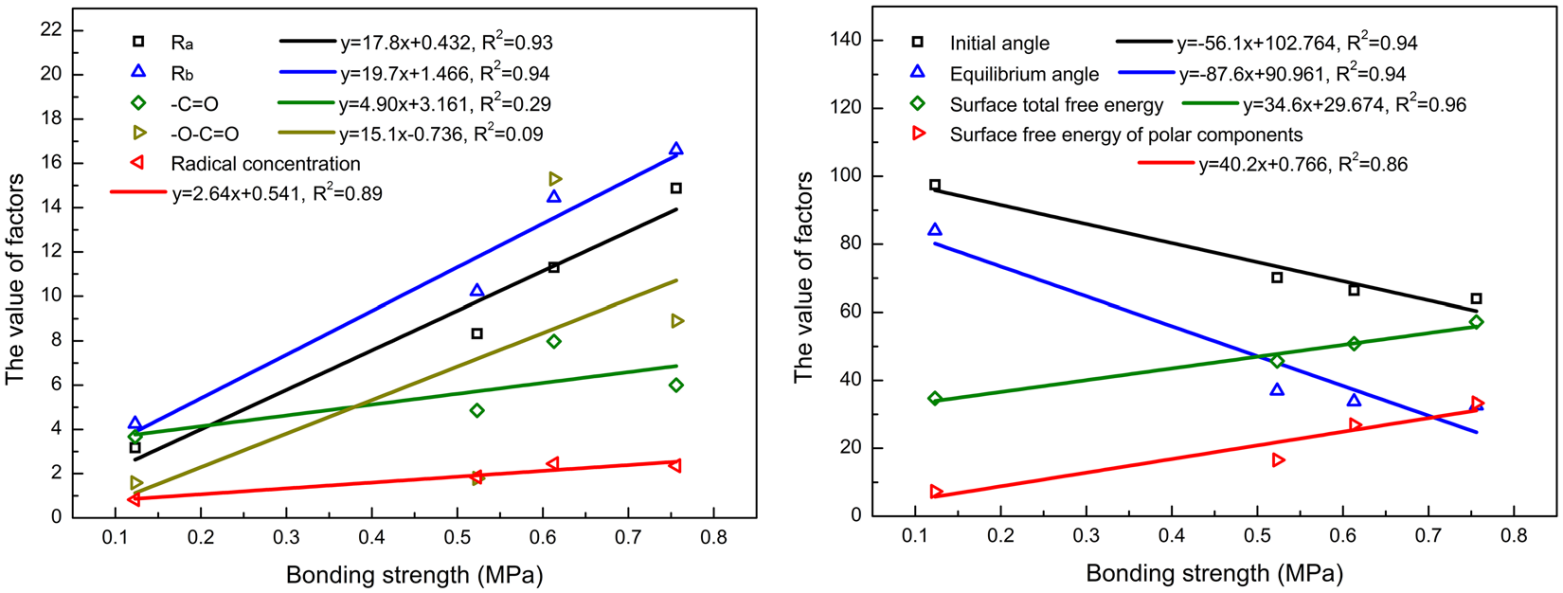
The linear relationships between bonding strength and physical and chemical properties.

To investigate the influence of contributing parameters on the bonding strength of modified samples, the relationships between the bonding strength of outer-inner WSC (using PF as adhesive) and surface roughness parameters (*R*_*a*_ and *R*_*b*_), surface chemical components (-C=O, -O-C=O, and free radicals), surface contact angles (initial and equilibrium angles), and surface free energy (surface total free energy and surface free energy of polar components) were evaluated as presented in Fig. 5. All the surface physical properties (*R*_*a*_, *R*_*b*_, initial, equilibrium angles, and surface total free energy) demonstrate a good linear relationship with bonding strength showing that the correlation coefficients are all higher than 0.90. However, the low correlation coefficients for the linear relationship between bonding strength and the surface chemical properties (-C=O and -O-C=O) are observed. Although excessive plasma discharge power can lead to the generation of more free radicals and oxygen-containing chemical groups, it results in self-degradation and gasification on WOS simultaneously. In this case, physical properties changes are more appropriate than the chemical properties to assess the bonding strength of WOS. Higher roughness and lower contact angle can make adhesive get more access to free radicals and introduced oxygen-containing chemical groups providing more contact area for PF or UF to form the nail-like adhesive after curing and thus improving the bonding strength of WSC. Therefore, water vapor plasma under moderate discharge power can fast enhance the bonding strength of WSC owing to its synergistically improving effects of both surface physical properties and chemical properties.

## Conclusion

Water vapor plasma leads to the appearance of surface roughness, the generation of massive free radicals, and the introduction of oxygen-containing groups. In addition, both initial and equilibrium contact angle and the surface total free energy were significantly increased after plasma modification. These results synergistically facilitate the spread and permeation of adhesive onto the wheat straw outer surface and thus improve the bonding strength of all prepared WSCs. In general, water vapor plasma is a time-saving and cost-effective modification method for the WSC manufacture. Also, this method is industrial applicable since the modified wheat straw can be randomly assembled whether in the form of outer-outer or outer-inner.

### Data availability

The data that support the findings of this study are available from the corresponding authors upon reasonable request.
